# Unplanned out-of-hospital birth and risk factors of adverse perinatal outcome: findings from a prospective cohort

**DOI:** 10.1186/s13049-019-0600-z

**Published:** 2019-03-02

**Authors:** François Javaudin, Valérie Hamel, Arnaud Legrand, Sybille Goddet, François Templier, Christine Potiron, Philippe Pes, Gilles Bagou, Emmanuel Montassier

**Affiliations:** 1 Department of Emergency Medicine, CHU Nantes, Nantes University Hospital, 44000 Nantes, France; 2 MiHAR lab, Université de Nantes, 44000 Nantes, France; 3 Emergency Department, Toulouse Purpan University Hospital, Toulouse, France; 4 DRCI, CHU Nantes, Nantes, France; 5 Samu-21, CHU de Dijon, SAU-Smur, CH du Creusot, Dijon, France; 6 Emergency Department, SAMU 49, University Hospital of Angers, Angers, France; 7 Samu, groupement hospitalier Édouard-Herriot, Lyon, France

**Keywords:** Emergency medical services, Out-of-hospital delivery, Out of hospital birth, Prehospital delivery, Birth before arrival, Unplanned delivery, Emergency medical services

## Abstract

**Background:**

In France, while most babies are delivered at hospital, emergency medical services (EMS) weekly manage calls for unplanned out-of-hospital births. The objective of our study was to describe neonatal morbidity and mortality, defined as death or neonatal intensive care unit hospitalization at Day 7, in a prospective multicentric cohort of unplanned out-of-hospital births.

**Methods:**

We prospectively analyzed out-of-hospital births from 25 prehospital EMS units in France. The primary outcome was neonatal morbidity and mortality, and the secondary outcome was risk factors associated with neonatal morbidity and mortality. A univariate logistic regression was first made, followed by a multivariate logistic regression with backward selection.

**Results:**

From October 2011 to August 2018, a total of 1670 unplanned out-of-hospital births were included. Of these, 1652 (99.2%) were singleton and 1537 (93.5%) had prenatal care. Maternal mean age of the study population was 30 ± 5.5 (range 15 to 48). The majority of women were multiparous, but 13% were nulliparous. Overall, 45.3% of these unplanned out-of-hospital births were medically-driven, either by phone during medical regulation (12.5%) or on scene by the prehospital emergency medical service units (32.9%). The prevalence of neonatal morbidity and mortality was 6.3% (*n* = 106) after an unplanned out-of-hospital birth (death before Day 7: *n* = 20; 1.2%). The multivariate logistic regression found that multiparity (adjusted Odds Ratio = 70.7 [4.7–1062]), prematurity (adjusted Odds Ratio = 6.7 [2.1–21.4]), maternal pathology (adjusted Odds Ratio = 2.8 [1.0–7.5]) and hypothermia (adjusted Odds Ratio = 2.8 [1.1–7.6]) were independent predictive factors of neonatal morbidity and mortality.

**Conclusions:**

Our study assessed for the first time risk factors for adverse perinatal outcome in a large and multicenter cohort of unplanned out-of-hospital births. We have to improve temperature management in the out-of-hospital field and future trials are required to investigate strategies to optimize newborns management in the prehospital area.

**Electronic supplementary material:**

The online version of this article (10.1186/s13049-019-0600-z) contains supplementary material, which is available to authorized users.

## Background

In France, while most babies are delivered at hospital, emergency medical services (EMS) weekly manage calls for unplanned out-of-hospital births [1–4]. Despite health system organizations variations, unplanned out-of-hospital birth can be defined as birth without midwife and medical care, or optimal health care conditions [5]. This specific context must be discriminated from planned out-of-hospital births, home births or freestanding birthing centers, where midwife management is performed [6]. Globally, unplanned out-of-hospital births prevalence is estimated to be 0.19 to 0.61% of all deliveries [7, 8].

Out-of-hospital delivery is associated with unfavorable perinatal outcome and increased mortality [9–11]. Hypothermia is the most frequently described adverse outcome [7, 8]. However, knowledge is limited by the small sample size of the previous studies, and most of them had insufficient power to accurately assess adverse events [12, 13]. Thus, perinatal outcome in unplanned out-of-hospital births remains unclear and large multicentric cohorts are needed to examine perinatal morbidity and mortality and their determinants.

The aim of our study was to describe neonatal morbidity and mortality, defined as death or neonatal intensive care unit hospitalization at Day 7, in a prospective multicentric cohort of unplanned out-of-hospital births.

## Methods

### Setting and Design of the Study

We designed a multicenter and prospective cohort of unplanned out-of-hospital births named AIE [14] (Observatoire des Accouchements Inopinés Extrahospitaliers; Out-of-hospital unexpected deliveries cohort), and conducted from October 2011 to august 2018. AIE involved 25 prehospital emergency medical service (EMS) units (Service d’Aide Médicale d’Urgence) among the 103 existing in France (24%) [15]. Population cared for by these units are reported in Additional file 1: Table S1. These units are ambulance base stations equipped with one or more mobile intensive care units, consisting of an ambulance driver, a nurse, and a senior emergency physician as the minimum team. In France, the EMS receive medical or trauma calls. Following protocols, the first dispatcher obtains the basic information from the caller, and then transfers the call to an emergency physician dispatcher, who performs a medical evaluation [15]. AIE was approved by the National Commission on Information and Freedom (CNIL/CCTIRS) and by French institutional review board. Maternal consent was systematically requested before or during birth management (left at the physician’s discretion) and data were continuously collected and anonymized at the end of follow-up on Day 7.

### Data collection

We registered a comprehensive description of the maternal demographic and health-related profile of women, the management by prehospital emergency medical service units and neonatal outcomes. Patient characteristics included age, date of birth, gestational age, gravidity, parity of the patient, abnormal blood glucose or gestational diabetes, mode of delivery and complications in previous pregnancies. We also collected data from the dispatch (time of call, the time of unit dispatched and the time of the unit reaching the patient, score predicting imminent delivery, the time of the unit reaching the hospital), the management in the prehospital emergency medical unit (active management of the delivery, non-instrumental obstetric maneuvers), the neonatal adaptation (Apgar score at 1 min, 5 min or on EMS arrival), the basic maternal hemodynamic measurements (pulse rate, blood pressure), and the adverse events (coiling of the umbilical cord, prolapse of the umbilical cord, dystocia of the shoulders, breech presentation, meconium stained amniotic fluid, hypothermia) [14]. We also collected data until Day 7 to assess the perinatal outcome of the newborns.

### Outcomes of the study

The primary outcome was neonatal morbidity and mortality in the cohort, and the secondary outcome was risk factors associated with neonatal morbidity and mortality.

### Statistical analysis

Continuous quantitative variables were categorized using tercile methods, and qualitative variables were encoded in term of absence/presence. Data were collected using Excel software (Microsoft Systems, Redmond, Washington, USA). Pearson’s Chi square tests, with Yates Correction when appropriate, were used to test association with the primary outcome for each variable, and univariate logistic regression was performed. Correlation and covariance matrices were systematically analyzed to identify variable interlinking and avoid data circularity. We then performed a multivariable logistic regression with the backward method. Continuous variables were analyzed by ANOVA and/or ANCOVA (adjustment on confounding factors) after test for normality with Liliefors test and homoscedasticity with Levene test. A *P*-value less than 0.05 was considered significant. Statistical analyses were performed using STATISTICA 12.0 software (Statsoft).

## Results

### Demographic and clinical profile of the population

From October 2011 to August 2018, a total of 1670 unplanned out-of-hospital births were included from 25 prehospital emergency medical service units in France. Of these, 1652 (99.2%) were singleton and 1537 (93.5%) had prenatal care. Maternal mean age of the study population was 30 ± 5.5 (range 15 to 48). The majority of women were multiparous, but 13% were nulliparous. Nine percent of the women included in the cohort had comorbidities and 7.5% had already experimented preterm births. Importantly, 347 (23.6%) women made a midwife consultation in the last 24 h before the unplanned out-of-hospital birth. To note, 193 women (11.7%) were documented as non-French speaking. Moreover, the great majority of the unplanned out-of-hospital births did not include a context of pregnancy or fetal pathology, as depicted in Table 1.

**Table 1:** AIE cohort characteristics

Characteristic	our Cohort (*N* = 1670)
EMS Caller Profile N (%)	
Woman	370 (22.2%)
Close One Person	977 (58.6%)
Midwife	32 (1.9%)
Fireman/Paramedic	118 (7.1%)
Liberal Physician	4 (0.2%)
Call Transfer Center	112 (6.7%)
Other (Care Center…)	55 (3.3%)
Women Age N (%)	
< 20 years	28 (1.8%)
20–34 years	1121 (72.9%)
≥ 35 years	389 (25.3%)
Pregnancy N (%)	
Singleton	1652 (99.2%)
Multiple	13 (0.8%)
Parity N (%)	
0	220 (13%)
1	743 (44.6%)
2	401 (24,1%)
≥ 3	302 (18.3%)
Antenatal Care N (%)	
Conform	1537 (93.5%)
None	47 (2.9%)
Denial or Unknown Pregnancy	59 (3.6%)
Pregnancy Pathology N (%)	
Yes	368 (22.2%)
No	1289 (77.8%)
Fetal Pathology N (%)	
Yes	16 (1%)
No	1634 (99%)
Midwife consultation in the last 24 h	
Yes	347 (23.6%)
No	1126 (76.4%)
Cephalic Presentation N (%)	
Yes	1552 (98.3%)
No	27 (1.7%)
Prematurity (Weeks to term)	
0–2	838 (67.5%)
2–4	304 (24.4%)
4–8	70 (5.7%)
> 8	30 (2.4%)
Neonates Characteristics (% or Mean ± SD (N))	
Girls/Boys (%)	53%/47% (1665)
Weight (g)	3008 ± 832 (1649)
APGAR M1	9 ± 2 (591)
APGAR M5	10 ± 1 (591)
APGAR at EMS arrival	10 ± 1 (1072)

*EMS* emergency medical services, *APGAR score* Activity, Pulse, Grimace, Appearance, Respiration, *SD* Standart Deviation

The majority of unplanned out-of-hospital births occurred at term, with an overall mean gestation of 38 weeks. There was a small proportion of babies (8.1%) born at less than 36 weeks gestation, including 30 (2.4%) who were very premature, that is under 32 weeks gestation. Unplanned out-of-hospital births mostly took place at home (78%), but 11.5% happened in ambulances and 3.8% in cars on the way to hospital. Overall, 45.3% of these unplanned out-of-hospital births were medically-driven, either by phone during medical regulation (12.5%) or on scene by the prehospital emergency medical service units (32.9%). We also found that 236 births (14.2%) were only accompanied by a rescuer. Moreover, in only 6 cases (0.4%), specialized neonatal prehospital EMS unit, managed by pediatric emergency physicians, had been sent to care for the newborn. Globally, 35.4% of unplanned out-of-hospital births in our cohort occurred without medical care. We also found that 14% (*n* = 83) of the newborns had an Apgar score scored ≤7 out of 10, and that mean Apgar score was 9 ± 2 at 1 min and 10 ± 1 at 5 min. We also observed that 58.0% of the unplanned out-of-hospital births occurred between 20.00 and 8.00.

### Neonatal morbidity and mortality

Neonatal morbidity and mortality, defined as death or neonatal intensive care unit (NICU) hospitalization at Day 7, was recorded in 106 newborns (6.3%). Mortality before the 7th day was 1.2% (*n* = 20). Neonatal morbidity and mortality emphasized a non-significant inter-annual variation, from 4.4% in 2012 to 9.2% in 2016 (*p* = 0.08). We also pinpoint significant inter-center difference, from 1.6 to 16.4% (*p* < 0.0001). We did not found significant difference in neonatal morbidity and mortality between unplanned out-of-hospital births with prehospital emergency medical service unit management and those without (5.3% versus 6.9%, *p* = 0.21).

Univariate regression logistic analysis highlighted that prenatal care, breech presentation, intercurrent physician/midwife consultation, primiparity, unhealthy house, midwife consultation during the last 24 h and meconium-stained amniotic fluid were risk factors of neonatal morbidity and mortality as defined in the primary outcome. The multivariate logistic regression found four predictive factors for neonatal morbidity and mortality: multiparity (adjusted Odds Ratio = 70.7 [4.7–1062]), prematurity (adjusted Odds Ratio = 6.7 [2.1–21.4]), maternal pathology (adjusted Odds Ratio = 2.8 [1.0–7.5]) and hypothermia (adjusted Odds Ratio = 2.8 [1.1–7.6]). They are reported in Table 2. Importantly, time of call, time of unit intervention and time of unit reaching the hospital, did not appear to be significantly associated with the risk of neonatal morbidity and mortality.

**Table 2:** Univariate and multivariate models of outcome

Variables	Neonatal mortality and morbidity ** *(N = 106)*	Safe Birth *(N = 1564)*	Chi2-Pearson *p*-value	Univariate Logistic Regression Odds-Ratio [CI 95%]	Multivariate Logistic Regression Adjusted Odds-Ratio [CI 95%]	*P*-value
Time of call *	34.3% (34/99)	31.6% (451/1427)	0.57			
Time of unit intervention *	37.1% (39/105)	34.1% (513/1503)	0.53			
Time of unit reaching the hospital (ie. > 30 min)*	21% (19/91)	19% (263/1417)	0.58			
Non-French speaking	8.6% (9/104)	12.0% (184/1535)	0.31			
Unhealthy housing	17.0% (14/82)	9.0% (113/1266)	0.02	2.1 [1.1–3.9]		
Multiple pregnancy	5.6% (6/108)	0.5% (7/1557)	< 0.0001	13.2 [4.4–40]	70.7 [4.7–1062]	0.002
Primiparity	35.0% (37/106)	12.0% (179/1548)	< 0.0001	4.1 [2.7–6.3]		
Intercurrent midwife or physician consultation	9.3% (10/108)	2.9% (46/1560)	< 0.001	3.4 [1.7–7]		
Conform antenatal care	81.1% (86/106)	94.2% (1451/1540)	< 0.0001	0.3 [0.2–0.4]		
Midwife consultation in the last 24 h	12.0% (11/93)	24.0% (333/1370)	0.006	0.4 [0.2–0.8]		
Older maternal age *	7.0% (34/106)	6.2% (65/1041)	0.57			
Fetal pathology	5.7% (6/106)	0.7% (10/1548)	< 0.0001	9.2 [3.2–26]		
Prematurity (ie. < 37 weeks of gestational age)	36.0% (38/105)	4.0% (60/1455)	< 0.0001	34.7 [19–63.5]	6.7 [2.1–21.4]	0.001
Maternal pathology	50.0% (53/106)	20.0% (314/1540)	< 0.0001	9.1 [3.3–25.7]	2.8 [1.0–7.5]	0.047
Breech presentation	7.0% (6/86)	1.4% (21/1493)	< 0.001	5.4 [2.1–13.7]		
Cord prolapse	15.4% (12/78)	18.9% (230/1217)	0.44			
Meconium-stained amniotic fluid	24.0% (15/63)	14.0% (123/886)	0.03	1.9 [1.1–3.6]		
Hypothermia	35.9% (19/53)	20.6% (184/892)	< 0.001	2.2 [1.2–3.9]	2.8 [1.1–7.6]	0.039

*Defined by extreme population tercile T3 versus T1+; ** Defined as death or neonatal intensive care unit hospitalization at Day 7; NICU: neonatal intensive care unit; CI 95%: confidence interval 95%

## Discussion

To the best of our knowledge, this is the first study that assessed neonatal mortality and morbidity, and risk factors for adverse perinatal outcome in a large and multicenter cohort of unplanned out-of-hospital births. Here, we found that mortality was rare in unplanned out-of-hospital births (1.2%), and that multiparity, prematurity, maternal pathology and hypothermia were independent predictive factors for poor neonatal outcome.

All emergency medical services (EMS) manage calls for unplanned out-of-hospital births ^1–4^. Prevalence is estimated to be 0.61% of all deliveries in the United States [7], 0.19 and 0.42% in two different studies in France, and 0.10% in Finland [3, 8, 11]. Most of previous works investigated out-of-hospital births without discriminating planned or unplanned births [16–21]. However, unplanned out-of-hospital births may be associated with higher unfavorable perinatal outcome and increased mortality [22]. By focusing on the management of unplanned births in the French healthcare system, AIE delivered for the first time epidemiological data powered for a robust analysis of perinatal morbidity and mortality outcome. Three previous studies reported unplanned out-of-hospital births management in small cohorts. McLelland et al. evaluated, in a retrospective data analysis collected via the Victorian Ambulance Clinical Information System, during a one year-period, 324 out-of-hospital births including 190 before paramedics’ arrival. In line with our results, mother had a mean age around 30 years and were at term. Similarly to our study, most of the births (88.3%) were uncomplicated births in multiparous women. Obstetric complications included postpartum hemorrhage (6.5%), breech (1.3%), cord prolapse (0.6%), and prematurity (11%) [23]. Scott and Esen reported 14 cases of unplanned out of hospital births, which occurred over a three-year period, with a reported incidence of 0.31% [24]. All the women were multiparous, without any context of maternal or fetal pathology. As described by us and McLelland et al. [23], most of the births (79%) occurred between the hours of 20.00 and 08.00 [24]. Moreover, Flanagan et al. reported in a retrospective analysis of 192 unplanned out-of-hospital births, that 21% of the newborns had an Apgar score scored ≤7 out of 10, whereas in our cohort, it represented only 14% of the newborns [25]. Contrary to previous reports, a considerable proportions of mothers in our cohort (23.6%) had a midwife consultation in the 24 previous hours. For instance, Flanagan et al. reported that only 2.4% (*n* = 15) of women who birthed before arrival stated that they had been sent home from hospital within the 12 previous hours [25].

In our study including only unplanned out-of-hospital births, neonatal morbidity and mortality, defined as death or neonatal intensive care unit (NICU) hospitalization at Day 7, was recorded in 106 newborns (6.3%). Neonatal mortality varies substantially between the different cohorts already reported. In a prospective case series of consecutive out-of-hospital deliveries in United States, Moscovitz et al. reported 9 neonatal deaths among 91 out-of-hospital deliveries (9.9%), with 86% occurring in the presence of paramedics [20]. McLelland reported nine (2.7%) neonatal deaths, including three that were not viable being less than 24 weeks gestation [23]. In France, Renesme et al. reported one neonatal death in a retrospective case–control study of 76 unplanned out-of-hospital births, and eleven admissions in NICU (14.5%) [3]. In a retrospective Finish cohort with 67 out-of-hospital births recorded, Ovaskainen et al. found that out-of-hospital cases were more likely to be admitted to the neonatal care unit and to be treated for suspected infections and hypothermia, even if no neonatal death was reported [11].

Risk factors for planned or unplanned out-of-hospital births have been widely explored, highlighting contribution of factors including ethnic group, age, parity, prenatal care, education, labour duration, smoking or distance to maternity center [3, 11, 12, 20]. Perinatal morbidity and mortality in planned and unplanned out-of-hospital births was associated with several factors, including postpartum hemorrhage, puerperal complications, low birth weight, polycythemia, and hypothermia [9, 10, 12, 13, 19]. Unsurprisingly, as reported in our cohort, prematurity worsens perinatal prognosis [21]. Moreover, in line with previous studies, our multivariate logistic regression model highlighted the impact of hypothermia in unplanned out-of-hospital births. A retrospective, monocentric case–control study in Israel of women who underwent unplanned home or car births (*n* = 90) versus in-hospital births (*n* = 180), already reported that significantly more newborns delivered out of the hospital had hypothermia [13]. Moscovitz et al. reported that hypothermia was common (47%) in the paramedic-attended deliveries [20], and hypothermia was reported in 50% of the newborns in another retrospective study [24]. Similarly, in another retrospective case-control study of unplanned out-of-hospital births (*n* = 81) in France, Renesme et al reported that NICU admission rate was increased in case of unplanned out-of-hospital births and that the most frequent complication was hypothermia [3].

Our results were in line with the literature but the strength of our study is to specially highlight the association between hypothermia and neonatal adverse outcome in a cohort of unplanned out-of-hospital births. Thus, we have here opportunities to improve the out-of-hospital management of unplanned births to limit the hypothermia of the newborn. We have to study the different tools we can use to keep the newborn warm. For instance, to warm the birthplace and keep if free from drafts, to place the newborn on the abdomen of the mother and dry him with a warm dry cloth and then leave the newborn on the abdomen of the mother (skin-to-skin contact) [26], or to systematically use an incubator device to closely monitor temperature. Moreover, we have to compare all these available tools and deliver recommendations to prevent the loss of body heat of the newborns on the out-of-hospital field. To date, the best option to reduce loss of body heat of the newborns during out-of-hospital management is unknown.

Importantly, lack of prenatal care or history of poor prenatal care was infrequent in our cohort (2.9%), and was not associated with neonatal mortality and morbidity as found in previous studies [20]. Multiple pregnancy, maternal pregnant pathology and prematurity were independently associated to adverse neonatal outcome. One option could be to systematically send a specialized neonatal prehospital EMS unit to care for the newborn when these risk factors are reported during the medical evaluation of the call by the emergency physician dispatcher. In France, such specialized units exist, managed by pediatric emergency physicians, and are equipped with materials dedicated for neonatal and premature care. Such specialized teams may efficiently take in charge neonatal distresses. Thus guidelines, randomized and controlled trials are required to investigate strategies to optimize newborns management in the prehospital area that could decrease neonatal morbidity and mortality [27]. Healthcare system should take into account these risk factors to give the appropriate care during the out-of-hospital phase.

## Limitations

Our study has several limitations. First, the main limitation of our study was that although AIE data are very complete for some variables, missing data and use of null values occur more often for others, as reported in Table 2. Filling of the case report forms was satisfying for emergency medicine research, but heterogeneous according tested variables. However, we are confident that this limitation did not affect the integrity of the study. Second, we have no data on the mothers who denied inclusion in the study as we were not able to perform an accurate and detailed screening process of all eligible mothers. Third, several tested variables were infrequent, resulting in large 95% confidence interval, as found in other studies [20]. Fourth, in France, mobile intensive care units consisted of an ambulance driver, a nurse, and a senior emergency physician as the minimum team. Thus, our practice may not be strictly comparable with the ones reported in the literature where out-of-hospital management is performed by paramedics.

## Conclusions

Our study assessed for the first time neonatal mortality and morbidity, and risk factors for adverse perinatal outcome in a large and multicenter cohort of unplanned out-of-hospital births. We found that multiparity, prematurity, maternal pathology and hypothermia were independent predictive factors for neonatal morbidity and mortality. We have to improve temperature management in the out-of-hospital field and future trials are required to investigate strategies to optimize newborns management in the prehospital area.

## Additional file

Additional file 1: **Table S1.** Population cared for by the 25 Emergency Medical Services involved in the study. (DOCX 63 kb)

## Abbreviations

AIE: Out-of-hospital unexpected deliveries cohort
EMS: emergency medical services
NICU: neonatal intensive care unit
