# Supplementary material for: Unplanned out-of-hospital birth and risk factors of adverse perinatal outcome: findings from a prospective cohort
Source: Scand J Trauma Resusc Emerg Med. 2019 Mar 2;27:26. doi: 10.1186/s13049-019-0600-z (PMC6397745; doi:10.1186/s13049-019-0600-z)
Supplement: Supplementary file 1 — Table S1. Population cared for by the 25 Emergency Medical Services involved in the study. (DOCX 63 kb) [file 13049_2019_600_MOESM1_ESM.docx]

**Additional file 1 : Table S1. Population cared for by the 25 Emergency Medical Services (EMS) involved in the study.**

|  | **2015** | **2016** |
| --- | --- | --- |
| EMS A | 152 730 | 153 067 |
| EMS B | 279 169 | 278 697 |
| EMS C | 533 147 | 533 213 |
| EMS D | 434 035 | 433 929 |
| EMS E | 1 335 103 | 1 348 183 |
| EMS F | 1 120 190 | 1 132 481 |
| EMS G | 1 251 060 | 1 252 912 |
| EMS H | 1 365 227 | 1 380 852 |
| EMS I | 173 400 | 173 347 |
| EMS J | 810 186 | 810 934 |
| EMS K | 1 044 486 | 1 045 271 |
| EMS L | 2 605 238 | 2 603 723 |
| EMS M | 228 582 | 227 829 |
| EMS N | 471 038 | 474 369 |
| EMS O | 1 370 678 | 1 381 249 |
| EMS P | 555 408 | 555 023 |
| EMS K | 568 445 | 567 561 |
| EMS R | 571 879 | 572 744 |
| EMS S | 386 543 | 386 448 |
| EMS T | 255 274 | 256 897 |
| EMS U | 557 548 | 559 014 |
| EMS V | 666 714 | 670 597 |
| EMS W | 1 276 233 | 1 287 330 |
| EMS X | 1 601 569 | 1 603 268 |
| EMS Y | 1 592 663 | 1 606 660 |
